# 17β-Estradiol Alters Rat Type-II Alveolar Cell Recovery from High Levels of Ozone

**DOI:** 10.1371/journal.pone.0090530

**Published:** 2014-03-05

**Authors:** Madeleine Chalfant, Karen K. Bernd

**Affiliations:** Department of Biology, Davidson College, Davidson, North Carolina, United States of America; University of Colorado, Denver, United States of America

## Abstract

Respiratory health is negatively impacted by exposure to ozone or to estrogens. Increasingly, individuals have simultaneous environmental exposure to both compounds. Characterizing the cellular responses stimulated by the combination of ozone and estrogens, therefore, is crucial to our complete understanding of the compounds' environmental health impacts. Our work introduces an alveolar cell culture model with defined media that provides evidence of ozone damage and determines sex hormones alter the cells' susceptibility to oxidative damage. Specifically, we investigated the individual and combined effects of environmentally relevant levels of ozone and 17β-estradiol on non-cancerous rat, type-II alveolar cells by examining biomarkers of cellular health and redox balance. The data reveal a complex role for 17β-estradiol in cellular recovery from 1 hr exposure to high ozone levels. At 0.5 hr post-ozone necrosis and inflammation markers show 17β-estradiol augments the detrimental effects of 350 ppb ozone, but after 24 hr of recovery, steroid treatment alters glutathione redox ratio and allows cellular proliferation.

## Introduction

Lungs encounter stressors, like ozone and estrogens, through simultaneous exposure to environmental and cellular sources including indoor and outdoor air, phytoestrogens and poly-aromatic hydrocarbons, and genetic makeup. Epidemiological analyses of the effects of exposure to higher ambient ozone concentrations have revealed a correlation with the incidence and severity of many lung pathologies including asthma [1], cancers [2], chronic obstructive pulmonary diseases (COPD), and pneumonia [3]. Cell-level studies of individuals with healthy or diseased lungs connect ozone exposure with acute and chronic pulmonary inflammation, with both types of inflammation postulated to be a part many lung disorders' pathogenesis [4]–[6]. When considering estrogens, compounds with both environmental routes of exposure and physiological differences due to genetic makeup and sex, there is agreement that the compounds alter lung pathogenesis. However, whether estrogens promote or inhibit disease remains in question ([7]–[9] *reviewed in* [10]–[12]). As indicated, previous research has focused on epidemiological or toxicological analysis of the individual effect of ozone or estrogen [13], [14] but, in the body, lungs are exposed to them simultaneously. An understanding how the combination of ozone and estrogen alters pulmonary pathogenic processes not only could help assess health risks posed by environmental exposure to estrogen and endocrine disrupting chemicals but also delineate the different health risks ozone may pose to male and female subpopulations, with the potential to inform intervention and treatment efforts.

Estrogens' cellular role is known to be complex. Because many cell types defend against oxidative damage by up-regulating antioxidant levels [1], the presence of additional 17β-estradiol (E2), the reduced and biologically active form of estrogen, might be predicted to mitigate ozone-induced damage. However, additional products of E2 metabolism increase the complexity of the potential cellular outcomes induced by ozonolysis. E2 exposed to oxidation is broken down [15] and many E2 metabolites increase ROS [16], alter redox homeostasis, and may be involved in carcinogenesis [17]. Compounding this effect, oxidation-induced upregulation of E2 levels may, in turn, upregulate cyclooxygenase-2 (COX-2) and increase expression of the inflammation marker, prostaglandin E2 (PGE2; [18])

In lung epithelia, although a connection between E2 and increased PGE2 has been described, PGE2's function is not well understood. In alveoli increased PGE2 decreases apoptosis and suppresses fibroblast proliferation; suggesting increased PGE2 prevents idiopathic pulmonary fibrosis and promotes lung health [19]. However, PGE2 is also associated with tumorigenesis [20], [21]. In type-II alveolar cells, because PGE2 alters the x_c_^−^ system for cystine transport, thereby decreasing cysteine available for synthesis of the antioxidant glutathione, PGE2 may also increase sensitivity to ROS [22]. A mechanism of E2 metabolism increasing ROS levels and PGE2 synthesis- which further augments ROS and thereby reduces antioxidant capacity and inhibits apoptosis- is consistent with E2's association with carcinogenesis and underscores the need for further investigation of the combined effects of E2 and strong oxidants like ozone.

The effect of E2 on recovery from oxidative stress is context dependent. E2 can affect recovery through plasma membrane-associated estrogen receptors (ERs) resulting in immediate alteration of signaling cascades, or via classical nuclear estrogen receptors, a slower, longer-lasting mechanism changing transcription rates. For example, E2 can inhibit glutathione synthesis via interactions with plasma membrane ERs that increase cAMP [23], [24]. However, E2 also increases expression of glutathione and enzymes in glutathione's biosynthetic pathway through nuclear ER-β in myocardial cells [25]. Because E2's effect on antioxidant levels is context dependent, research is needed determine how E2 alters non-cancerous cells' responses to oxidation.

Despite increased environmental exposure to ozone and estrogens and the individual correlations of each chemical to lung disease, the combined effect of ozone and estrogen on pulmonary health has not been examined in either whole animal or cell model systems. Due to the complexity of these variables' cellular effects, we developed a defined cell culture model focusing on the activation of cellular defense systems. Levels of well-established biomarkers were investigated to determine the effect of E2 and ozone, alone and in combination, on type-II alveolar cell health and redox homeostasis.

The survival of type-II alveolar cells during and after oxidative stress is critical to lung function. These cells participate in immune and inflammatory responses and, after lung injury, can proliferate and differentiate into type-I alveolar cells, the site of gas exchange [26]. We present a cell culture system using a non-cancerous, female rat type-II alveolar cell line (L2 cells; ATCC #CC-149) to characterize the combined effects of consistent exposure to physiologically relevant levels E2 (10 nM) and 1 hr exposure to Environmental Protection Agency (EPA)-defined ‘very unhealthy’ levels of ambient ozone (350 ppb). Separate recovery periods of 0.5 or 24 h hours allowed immediate and long-term responses to be evaluated. We assessed cellular health by determining relative levels of mitochondrial function, viability, necrosis, and apoptosis and by measuring the levels of total glutathione and glutathione disulfide (GSSG) and secreted PGE2.

## Materials and Methods

### Materials

Materials were obtained from the following suppliers: female rat non-cancerous type-II alveolar cells (CC-149, L2 cells) and fetal bovine serum (FBS), ATCC; low glucose DMEM, Hyclone. Phenol red-free low glucose DMEM, 17β-estradiol (E2) and 3,3′, 5-triiodo-L-thyronine sodium salt (T3), Sigma-Aldrich; charcoal-stripped FBS and 100X antibiotic-antimycotic, Invitrogen; Trypsin-EDTA .05%, VWR; Na-pyruvate, Cellgro; Hanks buffered saline solution (HBSS), Lonza; MTT, PGE2, and Apotox-Glo Triplex assay kits from Roche, Cayman Chemical, and Promega, respectively. Promega generously provided GSH/GSSG-Glo assay prior to public release.

### Cell culture and O_3_ exposure

L2 cells were cultured in a humidified atmosphere at 37°C, 5% CO_2_ in low glucose DMEM, 10% FBS. Cells were seeded into either white bottom or clear bottom 96-well, tissue culture treated plates (Costar) at 10^4^ cells/well. FBS contains uncharacterized levels of E2 and thyroid hormone (T3) and phenol red has been shown to have estrogen-like effects [34], therefore, after 18–24 hour attachment period, cells were washed (PBS) and *defined media* (phenol red-free, low glucose DMEM, 10% charcoal stripped FBS, 10^−9^ M T3) was added. Pretreatments (48 hours ±10 nM E2) occurred as indicated. To remove extracellular compounds that were oxidizable, cells were washed (PBS) and the media changed to HBSS ±10 nM E2 before gas exposure. Ozone was generated from O_2_ via an Ozone Gas Generator (Pacific Ozone Technology) and diluted to indicated concentrations with sterile 5% CO_2_/air. Exposure conditions included (2.5 L/min sterile 5% CO_2_/air) ±350 ppb O_3,_ 1 hr, 37°C. To isolate the effect of flowing air (itself a source of oxidation) versus non-flowing air, ‘No-flow’ (NF) samples, covered with parafilm, were included and used to normalize data as indicated. After gas exposure, cells were washed (PBS) and returned to defined media ±10 nM E2. Assays were performed 0.5-hour or 24-hour after gas exposure, as indicated.

### Biomarker assays

**Mitochondrial activity assay.** Quadruplicate assays determining mitochondrial activity via reductase activity (MTT assay) were performed as per manufacturer instructions (Roche). Absorbance values were measured spectrophotometrically (Model 680 Microplate Reader; Bio-Rad) with background readings (Abs_655nm_) subtracted from Abs_600nm_ readings. Data were normalized to the non-oxidized levels represented by average NF controls.

**Viability, necrosis and apoptosis assays.** Viability (GF-AFC cleavage), cytotoxicity (bis-AAF-R110 cleavage) and apoptosis (caspase 3/7 activity) were measured simultaneously via the Apotox-Glo™ Triplex assay (Promega). To facilitate collection of fluorescent and luminescent data cells were seeded into white-bottom 96 well plates. Quadruplicate samples were treated as indicated and processed per manufacturer instructions. An FLx800 Microplate Fluorescence Reader (Bio-Tek Instruments Inc.) was used to measure both fluorescence (420_Ex_/485_Em_ and 485_Ex_/528_Em_) and luminescence. Data were normalized to the non-oxidized levels represented by average NF samples for each subassay.

**Assay of inflammation marker.** Triplicate samples were seeded in clear-bottom 96 well plates, treated as indicated and PGE2 levels determined per manufacturer instructions (PGE2 Assay: Cayman Chemical Co.). Since fresh media was added after gas exposure, samples represent PGE2 secreted after oxidative stress. All samples were frozen (−80°C) immediately after collection and assays were performed within 2 weeks. PGE2 concentrations were calculated against concurrently run standards. The average concentration (pg/ml) is reported.

**Redox state.** Total glutathione and GSSG levels were used as a measure of the cells' redox state. Triplicate samples were prepared for each assay, treated as indicated, and processed per manufacturer instructions (*GSH/GSSG-Glo™ Assay* Promega). Total glutathione and GSSG concentrations were calculated against concurrently run standards and average µM for each condition is reported.

### Statistical analysis

Prior to norming, outliers were identified and removed from data sets by the Q-Test (90% confidence interval). Graphs present mean ± S.E.M. To compare the combined effects of ±350 ppb ozone and ±10 nM estrogen, pure model I two-way ANOVAs were performed followed by a Tukey HSD *post hoc* test (JMP statistical package, Cary, NC). p≤0.05 was considered significant.

## Results and Discussion

Despite increasing environmental exposure to ozone and estrogens and correlations between each chemical and lung disease, no studies in either whole animal or cell model systems have reported the combined effects of ozone and estrogen on pulmonary health. In addition, while alveoli play a critical role in lung function much about the stress and recovery response of these cells remains unknown. Here we introduce L2 cells as an alveolar cell culture system suitable for determining the effects of environmental pollutants. We support use of the L2 cell system by showing that, consistent with data from other animal and culture systems, ozone has deleterious effects. However, unlike other systems used for ozone research, our L2 cell system has defined estrogen levels allowing dual analysis of ozone and hormonal influences. Our data indicated that estrogen plays a complex role in response to an oxidative stress event with differences between immediate and more long-term outcomes.

In order to remain close to conditions found in the environment we exposed alveolar type II cells (L2 cells) to physiologically relevant concentrations of ozone and estrogen. More specifically, we exposed L2 cells to the ozone level classified by the EPA as ‘very unhealthy’ (350 ppb ozone/1 hr). We recognize that an experimental design with alveolar cells directly exposed to ozone differs from the *in vivo* situation where inspired gases react with respiratory tract tissue before reaching the alveolus. Therefore, the effective ozone exposure that our +350 ppb ozone samples experience is greater than those found in an alveolus of a whole lung respiring in a 350 ppb ozone environment. Given that alveolar ozone concentrations *in vivo* are neither available nor part of the EPA exposure definitions, we note this limitation of our model and submit that, compared to cell and whole animal studies with exposure parameters of 1000 ppb+ozone [27]–[29], the exposure level in our system better models environmental and physiological conditions.

Cells were exposed to the biologically active form of estrogen, 17β-estradiol (E2). Reports indicate that exposures to some, but not all, concentrations of E2 increase cell growth rate [32]. Because E2-induced changes in growth rate would confound comparative analyses of biomarkers, prior to characterizing the combined effect of E2 and ozone on L2, we tested the effect of 0, 1, 10, and 100 nM E2 and determined that 10 and 100 nM E2 do not change L2 growth rate (data not shown). The fact that 10 nM E2 does not increase is consistent with data from other systems [32]. Because 10 nM E2 is more physiologically relevant than 100 nM E2, 10 nM E2 exposures were used in this study.

Within the body, E2 can alter recovery from oxidative stress via immediate mechanisms affecting signaling cascades through plasma membrane-associated estrogen receptors (ERs) and via slower, longer-lasting genomic mechanisms affecting transcription through classical nuclear ERs [23]–[25]. Therefore to capture data within the immediate and genomic response mechanisms, we characterized our model system by measuring biomarkers for cellular health, inflammation and oxidative stress at 0.5 hr and 24 hr after gas exposure, respectively. To examine the combined effect of E2 and ozone, we exposed L2 cells for 1 hour to all permutations of 0 nM or 10 nM E2 plus 0 ppb or 350 ppb ozone (i.e. ±E2 ±O_3_) at a flow rate of 2.5 L/min.

**Figure 1:**
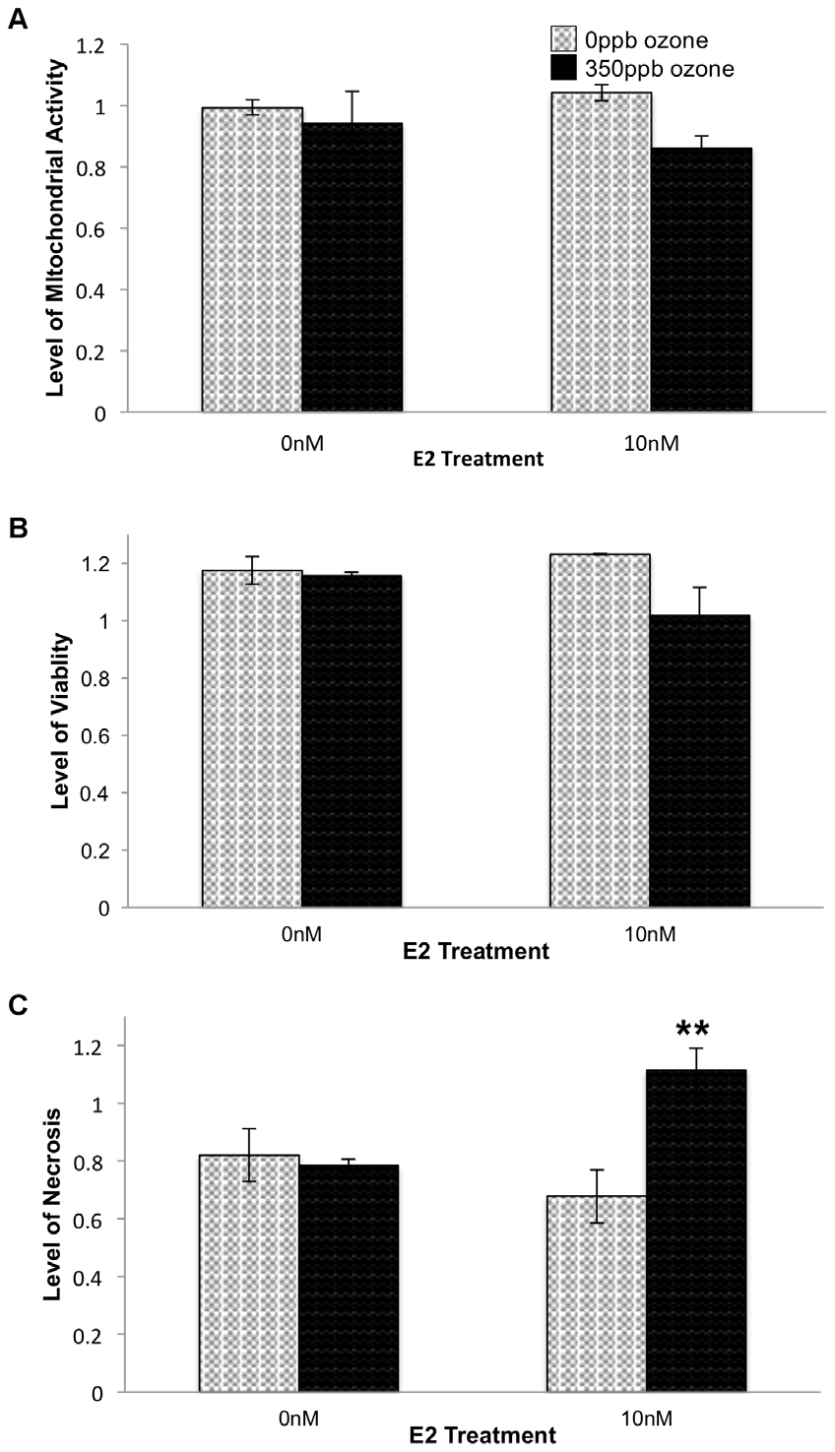
Effect of E2 and ozone (O_3_) on relative levels of mitochondrial activity (A), viability (B) and necrosis (C) 0.5 hr after gas exposure. 10^4^ L2 cells/well were treated with E2 and 350 ppb O_3_ as indicated. After 0.5 hr recovery time, levels of mitochondrial activity (F = 1.7336, df = 3,15, p = 0.2133), viability (F = 2.6161, df = 3,12, p = 0.1152), and necrosis (F = 7.4798, df = 3,12, p = 0.0081) were determined. Values represent the mean of 3–4 replicates normalized to data from control cells (-E2, in non-flowing 5% CO_2_/air), ±S.E.M. # p≤0.05 compared to 0 ppb O_3_. ** p≤0.01 compared to the same E2 treatment group.

First considering the 0.5 hr time point, exposure to 350 ppb ozone resulted in decreased viability and mitochondrial activity. Two-way ANOVA followed by Tukey HSD *post hoc* analysis revealed significant main effect of ozone, alone, on necrosis levels (p = 0.0113; Figure 1). 10 nM E2 treatment augmented ozone's effect and resulting in an additional statistically significant interaction effect and increase in necrosis (p = 0.0053; Figure 1).

**Figure 2:**
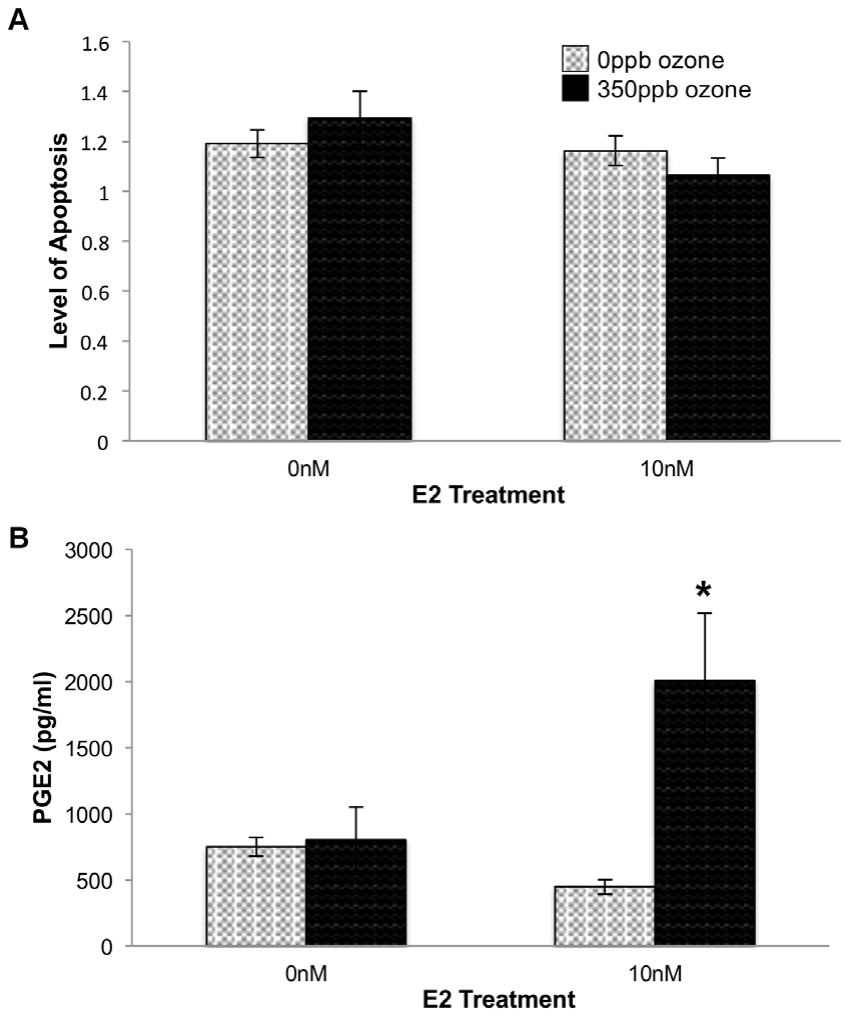
Effect of E2 and O_3_ on relative levels of apoptosis (A) and PGE2 secretion (B) 0.5 hr after gas exposure. 10^4^ L2 cells/well were treated with E2 and O_3_ as indicated. After 0.5 hr recovery time, levels of apoptosis (F = 0.6948, df = 3,12, p = 0.5781) were determined and media samples were collected from a separate set of cells to determine PGE2 secretion (F = 5.8628, df = 3,11, p = 0.0203). Level of apoptosis values represent the mean of 3–4 replicates normalized to data from control cells (-E2, in non-flowing 5% CO_2_/air), ±S.E.M. PGE2 values represent the mean of 3 replicates, ±S.E.M. # p≤0.05 compared to 0 ppb O_3_ group and * p≤0.05 compared to the same E2 treatment group.

Because ozone stimulates secretion of the inflammation marker, PGE2, and apoptotic enzyme activity and PGE2 levels show an inverse relationship in airway epithelia [30], we examined whether this relationship is maintained in L2 cells. Two-way ANOVA followed by Tukey HSD *post hoc* analysis revealed significant main effect of ozone on PGE2 secretion 0.5 hr post gas exposure (p = 0.0217) and an interaction effect between ozone and E2 resulting in an additional significant increase in PGE2 levels (p = 0.0295; Figure 2B). However, since neither ozone nor E2 altered activity of the apoptotic enzymes, Caspase 3/7, alveolar cells responds differently than airway epithelia and did not show the inverse relationship between PGE2 levels and apoptotic enzyme activity (Figure 2A).

**Figure 3:**
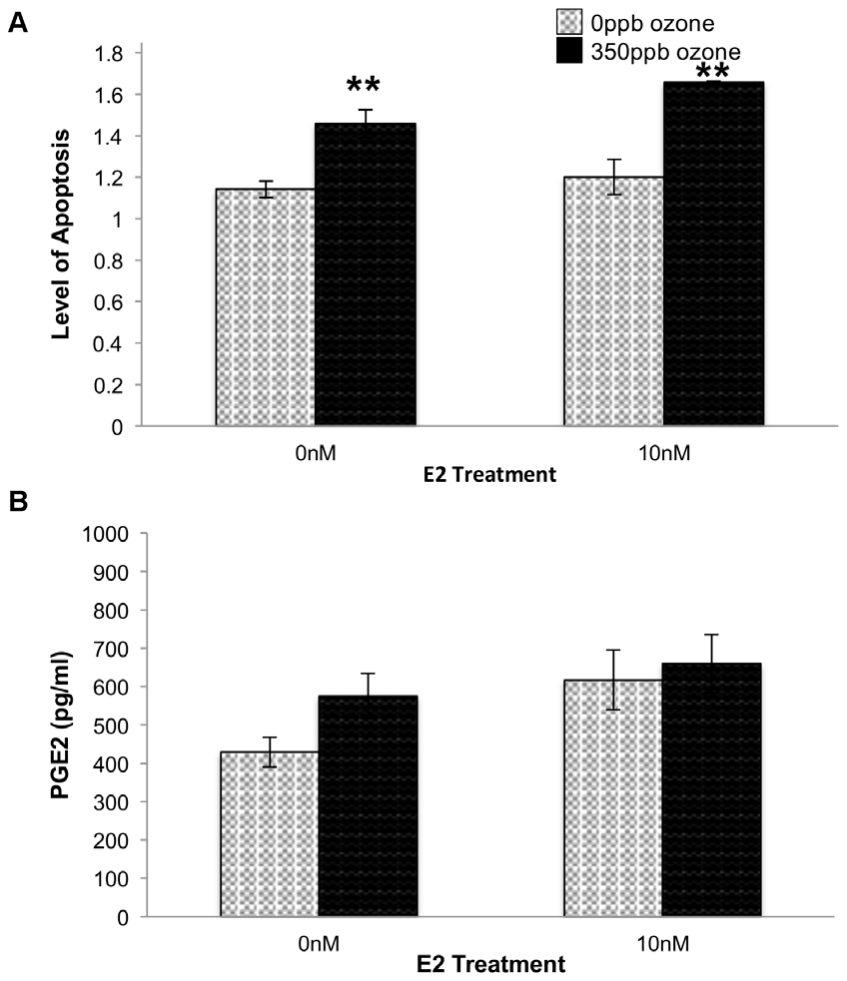
Effect of E2 and O_3_ on relative levels of apoptosis (A) and PGE2 secretion (B) 24 hr after gas exposure. 10^4^ L2 cells/well were treated with E2 and 350 ppb O_3_ as indicated. After 24 hr, levels of apoptosis (F = 12.0440, df = 3,13, p = 0.0012) were determined and media samples were collected from a separate set of cells to determine PGE2 secretion (F = 2.3803, df = 3,11, p = 0.1453). Values of apoptosis represent the mean of 3–4 replicates normalized to data from control cells (-E2, in non-flowing 5% CO_2_/air), ±S.E.M. ** p≤0.01 compared to 0 ppb O_3_.

**Figure 4:**
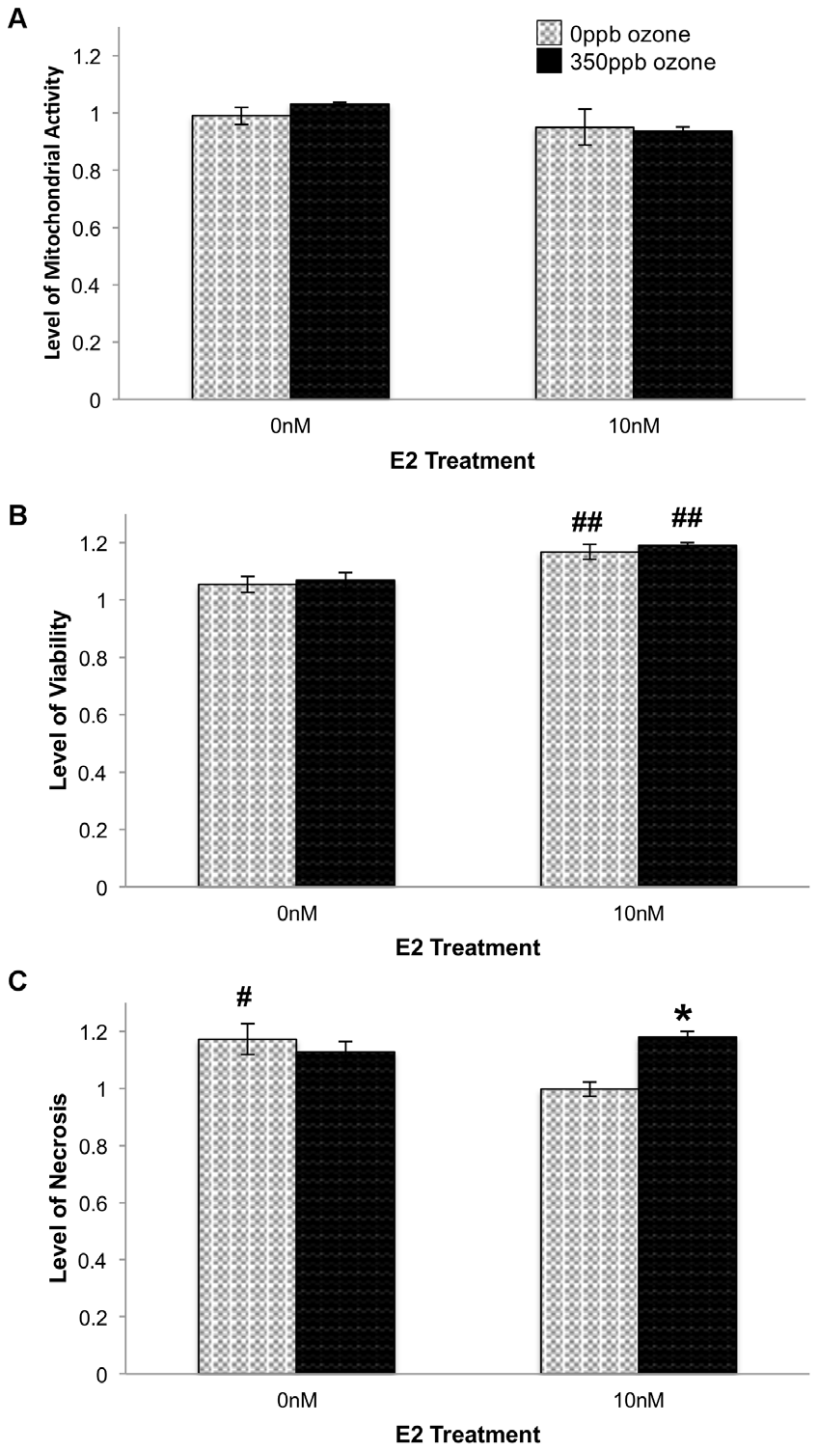
Effect of E2 and O_3_ on relative levels of mitochondrial activity (A), viability (B) and necrosis (C) 24 hr after gas exposure. 10^4^ L2 cells/well were treated with E2 and 350 ppb O_3_ as indicated. After 24 hr, levels of mitochondrial activity (F = 1.4122, df = 3,15, p = 0.2872), viability (F = 7.2915, df = 3,13, p = 0.0071) and necrosis (F = 5.9990, df = 3,13, p = 0.0132) were determined. Values represent the mean of 3–4 replicates normalized to data from control cells (-E2, in non-flowing 5% CO_2_/air), ±S.E.M.* p≤0.05 in the same E2 treatment group. # p≤0.05 and ## p≤0.01 compared to the same O_3_ exposure.

It has been suggested that an increase in PGE2 secretion may result in cell death through the generation of superoxide radicals [20], [31]. Thus, the additional increase in PGE2 seen in E2- treated samples at 0.5 hr post oxidative stress could be caused by a concomitant increase in ROS that stressed the cells to the point of irrevocable damage and necrosis. However, an interpretation of irrevocable damage would predict that at 24 hr post gas exposure, cell cultures treated with E2 would have fewer cells and therefore secrete less PGE2, than counterparts that were not treated with estrogen. In contrast, the data show that no combination of E2 and ozone significantly altered PGE2 secretion at the 24 hr time point (Figure 3B). In fact, in both 350 ppb ozone and 5%CO2/air treatment groups, including10 nM E2 resulted in an 11% increase in viability (p = 0.0009 Figure 4B).

Previous work demonstrated that type-II alveolar cells repair the alveolar wall after damage [27]. Consequently, we expected the increase in viability to be accompanied by an increase in cell proliferation that would, in turn, raise the sample's mitochondrial activity. Instead we found that E2 had no significant effect on mitochondrial activity in our alveolar cell system (Figure 4A) making our findings more similar to those of Si and colleagues [32] who reported 10 nM E2 did not induce aortic endothelia proliferation.

Because the increased viability seen in E2-treated samples could be the consequence of a decrease in necrotic cell death, a decrease in apoptotic death, or both, we subjected cells to all combinations of ±E2 and ±O_3_ and assayed necrosis and apoptosis biomarkers within the same sample. As predicted, samples treated with 10 nM E2 during and after 0 ppb ozone exposure (i.e. 2.5 L/min, 5% CO2/air) showed a significant decrease in necrosis when compared to cells exposed to flowing gas but no E2 (p<0.05; Figure 4C). In addition, the +E2-O_3_ samples exhibited significantly less necrosis than samples treated with E2+350 ppb ozone (p<0.05; Figure 4C). Comparing samples collected at the 0.5 hr and 24 hr post-gas recovery periods, necrosis in +E2+O_3_ samples increased by only 5.51% while in all other exposure conditions necrosis increased by 30–32% between the two time points (Figure 4C and 1C). This suggests that at the 24 hr time point a majority of the necrosis seen in the samples treated with E2+O_3_ reflects cell death that occurred immediately after gas exposure, rather than a significant increase in necrosis occurring *between* 0.5 and 24 hr of recovery. Examination of Caspase 3/7 activity levels revealed that, independent of E2 treatment, cells exposed to 350 ppb ozone showed significantly greater levels of apoptosis than cells exposed to 0 ppb ozone (p = 0.0002; Figure 3A). Taken together, these data suggest that when 10 nM E2 is present during a recovery period that is long enough to include changes in gene expression (i.e. 24 hr) the steroid mitigates ozone- induced necrosis, but not ozone-induced apoptosis.

Differences in study design and culture conditions could account for differences between previous studies and our cell proliferation data. First, earlier studies approximated cell growth via markers that occur before cytokinesis [27], [33] while our metrics required completion of cell division. Second, and perhaps more significantly, other studies, not focused on estrogens, used media containing phenol red, an estrogen mimic [34], and complete FBS containing undefined concentrations of E2. To decrease confounding media effects and better define E2 exposure levels in our system we cultured L2 cells in phenol-red free DMEM and 10% charcoal-stripped FBS. Thus, the increased cell proliferation in those other studies could be, in part, due to estrogenic effects of culture media. We find this hypothesis likely, as it is consistent with recent whole organism studies that report estrogen is responsible for some sex-specific differences in alveolar size [35] and is necessary for alveolar wall regeneration in mice [36]. Additionally, our initial feasibility studies showed that in the absence of any airflow 10 nM E2 treatment did not increase viability (Chalfant and Bernd, unpublished data). 10 nM E2 only increased viability when cells were under conditions more similar to those in the lung where low or high oxidative stress is present. These conditions are modeled in our system by 2.5 L/min, 5% CO2/air containing 0 ppb or 350 ppb ozone.

**Figure 5:**
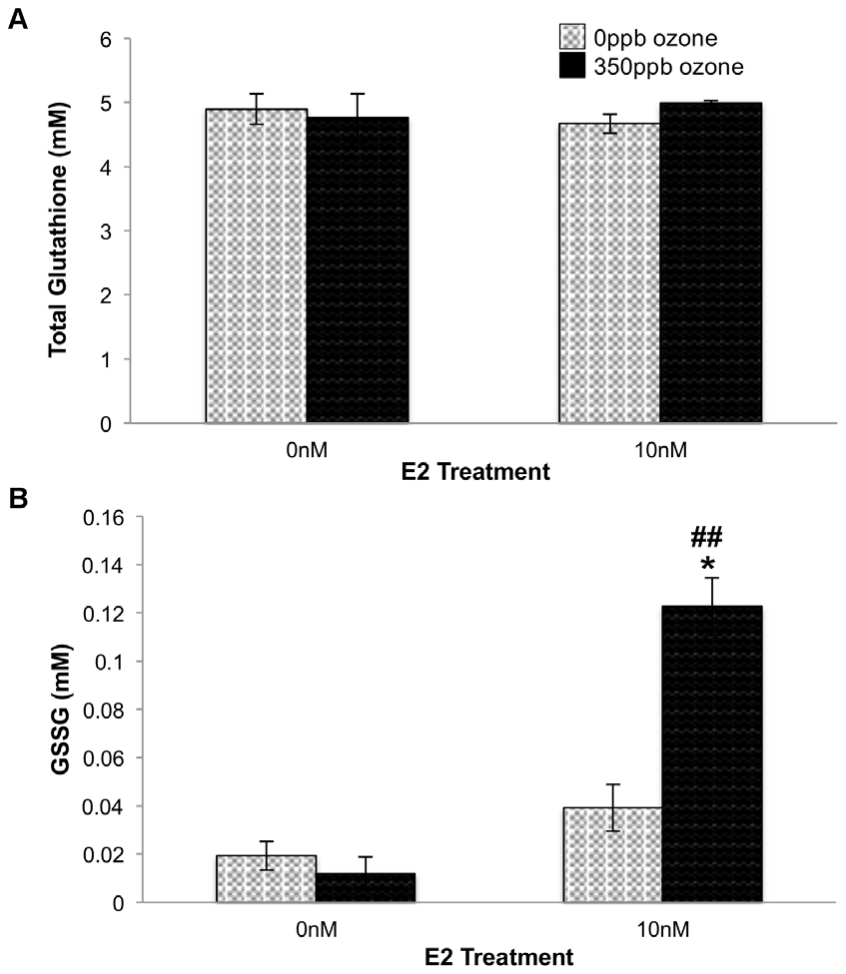
Effect of E2 and O_3_ on total glutathione (A) and GSSG (B) 24 hr after gas exposure. 10^4^ L2 cells/well were treated with E2 and 350 ppb O_3_ as indicated. After 24 hr recovery time total glutathione (F = 0.3699, df = 3,11, p = 0.7770) and GSSG (F = 33.0667, df = 3,11, p<0.0001) levels were determined. Values represent the mean of 3 replicates, ±S.E.M. ** p≤0.01 relative to the same E2 treatment. ## p≤0.01 same O_3_ exposure.

The increase in viability seen in E2-treated cells could occur by two different mechanisms. First, because E2 is involved in alveolar wall repair [36], it could induce proliferation that compensates for cell death despite high intracellular ROS. Conversely, viable cell number could be maintained because E2 could decrease damage caused by cellular ROS, either directly by acting as an antioxidant, or indirectly by increasing expression of the antioxidant glutathione. To explore the effect of E2 on the expression of cellular antioxidants, we measured total glutathione and found no significant difference between any combination of E2 and ozone treatments (Figure 5A). We note that assay limitations preclude determining cell number within the actual test population, thus, conditions that decrease cell numbers but increase glutathione per cell could show no net change in total glutathione. However, our data indicate that E2 treatment *increases* the number of viable cells in +350 ppb O_3_ conditions (Figure 4). Because we see no increase in glutathione in any condition tested, including those with increased viability, we are confident that E2's function in +350 ppb O_3_ conditions does not include upregulation of glutathione expression. Further investigation determined that ozone exposure (p = 0.0026) and E2 treatment (p<0.0001) significantly increased the amount of glutathione found in its oxidized form, GSSG (Figure 5B). In addition, together the two treatments synergistically increase GSSG levels in +E2+O_3_ vs. -E2- O_3_ controls (p = 0.0009). These data suggest that E2 increases ROS and enhances ozone-induced increases in ROS resulting in an altered glutathione redox ratio.

While E2 treatment increased cell viability after either low or high levels of oxidative stress, E2's effect on necrosis appears dependent upon the degree of oxidative stress in the system. E2-treated cells exposed to 0 ppb ozone had significantly lower levels of necrosis than those exposed to 0 ppb ozone without E2. Also, E2-treated cells exposed to 350 ppb O_3_ showed comparable high levels of necrosis at 0.5 hr and 24 hr post gas exposure, suggesting that while E2 exacerbated the original oxidative insult, the hormone eventually mitigated further damage to the cells. While this result could be due to increased levels of apoptosis, given cell proliferation seen in these conditions, we find that explanation unlikely.

As mentioned earlier, E2 could mitigate necrosis through several different mechanisms. E2 could act directly as an antioxidant, decreasing intracellular ROS and thus allowing recovery [37]. However this hypothesis is unlikely because the raised level of GSSG seen in 10 nM E2, 350 ppb ozone conditions supports the presence of high ROS. Other mechanisms that warrant exploration in future studies include E2 reducing ATP depletion caused by oxidative stress [38] and enabling ATP secretion that, in lung epithelia, is known to decrease ozone induced necrosis and apoptosis [39]. Alternatively, several studies in non-lung cell types have shown that E2 treatment increases basal levels of heat shock proteins (HSPs), which could play a role in recovery [40]–[42]. These reports, combined with those showing ozone stimulates expression of HSPs and stress proteins in type-II alveolar cells [27], [43], suggest that E2 treatment could enable cell survival by either increasing basal levels of HSPs or by further enhancing increases in HSP expression that ozone has induced.

The increase in cellular proliferation and reduction in cell death in the presence of increased ROS suggests that ozone exposure may induce E2 metabolism to one of its less understood metabolites. Both 2-hydroxyestradiol and 4-hydroxyestradiol increase cellular ROS and are known to induce DNA damage directly and via quinone – semi-quinone redox cycling [17]. Despite increasing intracellular ROS and DNA damage, 4-hydroxyestradiol also induces cell growth, providing a connection between E2 metabolism and cellular proliferation [16]. In our system E2 metabolism could be stimulated by tryptophan oxidized during ozone exposure. In mouse heptocarcinoma cells, AhR was activated both by oxidized tryptophan [44] and 2,3,7,8- tetrachlorodibenzo-p-dioxin (TCDD), the prototypical AhR ligand [45], resulting in increased expression of proteins involved in E2 metabolism [16]. In human bronchial epithelial cells, TCDD-AhR interactions were linked to induced expression of E2 metabolizing enzymes, decreased E2 and increased levels of its metabolites, 2-hydroxyestradiol and 4-hydroxyestradiol [46]. However while both metabolites are associated with increased ROS and cell growth, they also increase PGE2 secretion and decrease apoptosis [47]. Since, in our system, E2 treatment neither altered apoptosis levels in ozone treatments nor affected PGE2 secretion, it is important to continue examining other potential mechanisms.

In summary, we present a novel alveolar type II cell culture model that uses defined media conditions allowing characterization of simultaneous exposure to estrogen and ozone. Using this culture model we provide evidence that ozone and E2 treatments alter alveolar type-II cellular health metrics, both independently and in concert with one another. Our data suggest ozone significantly decreases viability, immediately causing necrosis and eventually increasing apoptosis. E2 treatment augments some of ozone's deleterious effects, increasing PGE2 secretion and increasing GSSG levels 0.5 hr and 24 hr after ozone exposure, respectively. However, E2 mitigates ozone's other effects, resulting in increased viability 24 hr post gas exposure. Our research provides greater insight into cellular mechanisms involved in sex differences in lung diseases and the effects of ozone exposure. While these topics are far from being understood, it is clear that ozone causes pulmonary damage and that sex hormones alter susceptibility to oxidative damage. Because E2 levels in the body vary and individual's environmental exposure to estrogens is increasing, our work underscores the need for further research to determine the extent of these trends.

## References

[1] Nadadur SS, Costa DL, Slade R, Silbjoris R, Hatch GE (2005) Acute ozone-induced differential gene expression profiles in rat lung. Environ Health Perspect 113: 1717–1722. 16330353 10.1289/ehp.7413 PMC1314911

[2] Dhondt S, Beck C, Degraeuwe B, Lefebvre W, Kochan B, et al (2012) Health impact assessment of air pollution using dynamic exposure profile: Implications for exposure and health impact estimates. Environ Impact Assess Rev 36: 42.

[3] Medina-Ramon M, Zanobetti A, Schwartz J (2006) The effect of ozone and PM10 on hospital admissions for pneumonia and chronic obstructive pulmonary disease: A national multicity study. Am J Epidemiol 163: 579–588. 16443803 10.1093/aje/kwj078

[4] Park GY, Christman JW (2006) Involvement of cyclooxygenase-2 and prostaglandins in the molecular pathogenesis of inflammatory lung diseases. Am J Physiol Lung Cell Mol 209: 797–805. 10.1152/ajplung.00513.2005 PMC4358817 16603593

[5] Rahman I (1999) Inflammation and the regulation of glutathione level in lung epithelial cells. Antiox Redox Signal 1: 425. 10.1089/ars.1999.1.4-425 11233143

[6] Klaunig JE, Kamendulis LM, Hocevar BA (2010) Oxidative stress and oxidative damage in carcinogenesis. Toxicol Pathol 38: 96–109. 20019356 10.1177/0192623309356453

[7] Dougherty SM, Mazhawidza W, Bohn AR, Robinson KA, Mattingly KA, et al (2006) Gender difference in the activity but not expression of estrogen receptors α and β in human lung adenocarcinoma cells. Endocrine Related Cancer 13: 113. 16601283 10.1677/erc.1.01118 PMC1472635

[8] Karlsson C, Helenius G, Fernandes O, Karlsson MG (2012) Oestrogen receptor beta in NSCLC - prevalence, proliferative influence, prognostic impact, and smoking. Acta Pathol Microbiol Immunol Scand 120: 451–458. 10.1111/j.1600-0463.2011.02850.x 22583357

[9] Cook MB, McGlynn KA, Devesa SS, Freedman ND, Anderson WF (2011) Sex disparities in cancer mortality and survival. Cancer Epidemiol Biomarkers 20: 1629–1637. 10.1158/1055-9965.EPI-11-0246 PMC3153584 21750167

[10] Verma MK, Miki Y, Sasano H (2011) Sex steroid receptors in human lung diseases. J Steroid Biochem Mol Biol 127: 216–222. 21856418 10.1016/j.jsbmb.2011.07.013

[11] Dransfield MT, Washko GR, Foreman MG, Estepar RSJ, Reilly J, et al (2007) Gender differences in the severity of CT emphysema in COPD. Chest 132: 464–470. 17573503 10.1378/chest.07-0863

[12] de Torres JP, Cote CG, Lopex MV, Casanova C, Diaz O, et al (2009) Sex differences in mortality in patients with COPD. Eur Respir J 33: 528–535. 19047315 10.1183/09031936.00096108

[13] Diamanti-Kandarakis E, Bourguignon J, Guidice LC, Hauser R, Prins GS, et al (2009) Endocrine-disrupting chemicals: An endocrine society scientific statement. Endocr Rev 30: 293. 19502515 10.1210/er.2009-0002 PMC2726844

[14] Fucic A, Gamulin M, Ferencic Z, Rokotov DS, Katic J, et al (2010) Lung cancer and environmental chemical exposure: A review of our current state of knowledge with reference to the role of hormones and hormone receptors as an increased risk factor for developing lung cancer in man. Toxicol Pathol 38: 869. 10.1177/0192623310378136 20805318

[15] Sindhu RK, Kikkawa Y (1999) Superinduction of oxidized tryptophan-inducible cytochrome P450 1A1 by cycloheximide in hepa 1c1c7 cell. In Vitro and Mol Toxicol 12: 149–162. 10894765

[16] Chang LW, Chang Y, Ho C, Tsai M, Lin P (2007) Increase of carcinogenic risk via enhancement of cyclooxygenase-2 expression and hyroxyestradiol accumulation in human lung cells as a result of interaction between BaP and 17-beta estradiol. Carcinogenesis. 10.1093/carcin/bgm013 17272310

[17] Roy D, Cai Q, Felty Q, Narayan S (2007) Estrogen-induced generation of reactive oxygen and nitrogen species, gene damage, and estrogen-dependent cancers. J Toxicol Environ Health 10: 235–257. 10.1080/15287390600974924 17620201

[18] Ho C, Ling Y, Chang LW, Tsai M, Lin P (2008) 17-beta estradiol and hydroxyestradiols interact via the NF-kappa B pathway to elevate cyclooxygenase 2 expression and prostaglandin E2 secretion in human bronchial epithelial cells. Toxicol Sci 104: 294–302. 18480072 10.1093/toxsci/kfn096

[19] Maher TM, Evans IC, Bottoms SE, Mercer PF, Thorley AJ, et al (2010) Diminished prostaglandin E2 contributes to the apoptosis paradox in idiopathic pulmonary fibrosis. American Journal of Critical Care Medicine 182: 73–82. 10.1164/rccm.200905-0674OC PMC2902759 20203246

[20] Greenhough A, Smartt HJM, Moore AE, Roberts HR, Williams AC, et al (2009) The COX-2/PGE2 pathway: Key roles in the hallmarks of cancer and adaptation to the tumour microenvironment. Carcinogenesis 30: 377. 19136477 10.1093/carcin/bgp014

[21] Klaunig JE, Kamendulis LM, Hocevar BA (2010) Oxidative stress and oxidative damage in carcinogenesis. Toxicol Pathol 38: 96–109. 20019356 10.1177/0192623309356453

[22] van de Wetering JK, van Golde LM, Batenburg JJ (2004) Collectins: Players of the innate immune system. Eur J Biochem 271: 1229–1249. 15030473 10.1111/j.1432-1033.2004.04040.x

[23] Bjornstrom L, Sjoberg M (2005) Mechanisms of estrogen receptor signaling: Convergence of genomic and nongenomic actions on target genes. Mol Endocrinol 19: 833–842. 15695368 10.1210/me.2004-0486

[24] Lu SC, Kuhlenkamp J, Garcia-Ruiz C, Kaplowitz N (1991) Hormone-mediated down-regulation of hepatic glutathione synthesis in the rat. J Clin Invest 88: 260–269. 1647417 10.1172/JCI115286 PMC296028

[25] Urata Y, Ihara Y, Murata H, Goto S, Koji T, et al (2006) 17β-estradiol protects against oxidative stress-induced cell death through the glutathione/glutaredoxin-dependent redox regulation of akt in myocardiac H9c2 cells. JBC 281: 13092–13102. 10.1074/jbc.M601984200 16549430

[26] Wang G, Umstead TM, Phelps DS, Al-Mondhiry H, Floros J (2002) The effect of ozone exposure on the ability of human surfactant protein A variants to stimulate cytokine production. Environ Health Perspect 110: 79–84. 11781168 10.1289/ehp.0211079 PMC1240696

[27] Wang J, Wang S, Manzer R, McConville G, Mason RJ (2006) Ozone induces oxidative stress in rat alveolar type II and type I-like cells. Free Radic Biol Med 40: 1914–1928. 16716893 10.1016/j.freeradbiomed.2006.01.017

[28] Funabashi H, Shima M, Kuwaki T, Hiroshima K, Kuriyama T (2004) Effects of repeated ozone exposure on pulmonary function and bronchial responsiveness in mice sensitized with ovalbumin. Toxicol 204: 75–83. 10.1016/j.tox.2004.06.047 15369850

[29] Last JA, Gohil K, Mathrani VC, Kenyon NJ (2005) Systemic responses to inhaled ozone in mice: Cachexia and down-regulation of liver xenobiotic metabolizing genes. Toxicol Appl Pharmacol 208: 117–126. 16183385 10.1016/j.taap.2005.02.001

[30] Kafoury RM, Hernandez JM, Lasky JA, Toscano WA, Friedman M (2007) Activation of transcription factor IL-6 (NF-IL-6) and nuclear factor-kappa B by lipid ozonation productions is crucial to interleukin-8 gene expression in human airway epithelial cells. Environ Toxicol 22: 159–168. 17366569 10.1002/tox.20246

[31] Klaunig JE, Kamendulis LM, Hocevar BA (2010) Oxidative stress and oxidative damage in carcinogenesis. Toxicol Pathol 38: 96–109. 20019356 10.1177/0192623309356453

[32] Si M, Al-Sharafi B, Lai C, Khardori P, Chang C, et al (2001) Gender difference in cytoprotection induced by estrogen on female and male bovine aortic endothelial cells. Endocrine 15: 255–262. 11762696 10.1385/ENDO:15:3:255

[33] Prokhorova S, Patel N, Laskin DL (1998) Regulation of alveolar macrophage and type II cell DNA synthesis: Effects of ozone inhalation. Am J Physiol Lung Cell Mol 275: LI200–LI207. 10.1152/ajplung.1998.275.6.L1200 9843858

[34] Welshons WV, Wolf MF, Murphy CS, Jordan VC (1988) Estrogenic activity of phenol red. Mol Cell Endocrinol 57: 169. 3402660 10.1016/0303-7207(88)90072-x

[35] Carey MA, Card JW, Voltz JW, Germolec DR, Korach KS, et al (2007) The impact of the sex and sex hormones on lung physiology and disease: Lessons from animal studies. Am J Physiol Lung Cell Mol 293: 272–278. 10.1152/ajplung.00174.2007 17575008

[36] Massaro D, Clerch LB, DeCarlo Massaro G (2007) Estrogen receptor-alpha regulates pulmonary alveolar loss and regeneration in female mince: Morphometric and gene expression studies. Am J Physiol Lung Cell Mol 293: L222–L228. 10.1152/ajplung.00384.2006 17449797

[37] Miyacuchi C, Muranaka S, Kanno T, Fujita H, Akiyama J, et al (2004) 17β-estradiol suppresses ROS-induced apoptosis of CHO cells through inhibition of lipid peroxidation-couples membrane permeability transition. Physiol Chem Phys Med NMR 36: 21–35. 15789971

[38] De Marinis E, Ascenzi P, Pellegrini M, Galluzzo P, Bulzomi P, et al (2010) 17beta-estradiol - A new modulator of neuroglobin levels in neurons: Role in neuroprotection against H2O2 toxicity. Neurosignals 18: 223–235. 21335947 10.1159/000323906

[39] Ahmad S, Ahmad A, McConville G, Schneider BK, Allen CB, et al (2005) Lung epithelial cells release ATP during ozone exposure: Signaling for cell survival. Free Radic Biol Med 39: 213–226. 15964513 10.1016/j.freeradbiomed.2005.03.009

[40] Zhang Y, Champagne N, Beitel LK, Gooodyer CG, Trifiro M, et al (2004) Estrogen and androgen protection of human neurons against intracellular amyloid beta toxicity through heat shock protein 70. J Neuroscience 24: 5315–5321. 10.1523/JNEUROSCI.0913-04.2004 PMC6729301 15190103

[41] Hamilton KL, Mbai FN, Gupta S, Knowlton AA (2004) Estrogen, heat shock proteins, and NF-kappaB in human vascular endothelium. Arterioscler Thromb Vasc Biol 24: 1628–1633. 15231513 10.1161/01.ATV.0000137188.76195.fb

[42] Porter W, Wang F, Duan R, Qin C, Castro-Rivera E, et al (2001) Transcriptional activaiton of heat shock protein 27 gene expression by 17beta-estradiol and modulation by antiestrogens and aryl hydrocarbon receptor agonists. J Mol Endocrinol 26: 31–42. 11174852 10.1677/jme.0.0260031

[43] Kosmider B, Loader JE, Murphy RC, Mason RJ (2010) Apoptosis induced by ozone and oxysterols in human alveolar epithelial cells. Free Radic Biol Med 48: 1513. 20219673 10.1016/j.freeradbiomed.2010.02.032 PMC2965594

[44] Sindhu RK, Mitsuhashi M, Kikkawa Y (2000) Induction of cytochrome P-410 1A2 by oxidized tryptophan in hepa 1c1c7 cells. J Pharmacol Exp Ther 292: 1008–1014. 10688617

[45] Singhal R, Shankar K, Badger TM, Ronis MJ (2008) Estrogenic status modulates aryl hydrocarbon receptor-mediated hepatic gene expression and carcinogenicity. Carcinogenesis 29: 227–236. 18174242 10.1093/carcin/bgm288

[46] Lin P, Chang Y, Chen C, Yang W, Cheng Y, et al (2004) A comparative study on the effects of 2,3,7,8-tetrachlorodibenzo-p-dioxin polychlorinated biphenyl126 and estrogen in human bronchial epithelial cells. Toxicol Appl Pharmacol 195: 83–91. 14962508 10.1016/j.taap.2003.11.001

[47] Ho C, Ling Y, Chang LW, Tsai M, Lin P (2008) 17-beta estradiol and hydroxyestradiols interact via the NF-kappa B pathway to elevate cyclooxygenase 2 expression and prostaglandin E2 secretion in human bronchial epithelial cells. Toxicol Sci 104: 294–302. 18480072 10.1093/toxsci/kfn096

